# The microbiome–inflammasome axis in endometriosis-associated chronic pelvic pain: mechanisms and therapeutic opportunities from an immunological perspective

**DOI:** 10.3389/fimmu.2026.1942637

**Published:** 2026-09-16

**Authors:** Xia Ma, Kang Lin, Yiqun Wang, Yi Xu

**Affiliations:** Department of Obstetrics and Gynecology, Taizhou Hospital of Zhejiang Province affiliated to Wenzhou Medical University, Taizhou, China

**Keywords:** dysbiosis, endometriosis, immunotherapy, innate immunity, NF-kappa B, NLRP3 inflammasome, pelvic pain, toll-like receptor 4

## Abstract

**Introduction:**

Endometriosis (EMs) is increasingly recognized as a chronic inflammatory disease in which innate immune dysregulation plays an important role. Its immunopathological features include macrophage polarization imbalance, with M1-skewed activation in inflamed peritoneal fluid and lesions, reduced natural killer (NK) cell cytotoxicity, adaptive immune alterations including Th2/Treg-skewed profiles reported in some studies, and persistently elevated pro-inflammatory cytokines. The disease affects approximately 10% of women of reproductive age worldwide, and 70%–80% of these patients experience chronic pelvic pain (CPP). Conventional hormonal therapies provide limited relief and are associated with high recurrence rates after withdrawal, yet the immunological drivers of CPP remain incompletely understood.

**Methods:**

We conducted a narrative review of peer-reviewed literature identified through PubMed, Web of Science, and Scopus up to August 2026. Search terms covered endometriosis, chronic pelvic pain, microbiota/dysbiosis, Toll-like receptor 4, NF-κB, NLRP3 inflammasome, IL-1β/IL-18, neuroinflammation, and immunotherapy. Reference lists were screened for additional relevant studies, and evidence was synthesized by mechanistic theme and appraised according to study design, directness, and consistency.

**Results:**

Convergent preclinical and observational evidence suggests that dysbiosis of the gut and reproductive tract microbiota may activate TLR4 via lipopolysaccharide, triggering NF-κB signaling and promoting NLRP3 inflammasome priming and activation. This cascade may contribute to M1-skewed pelvic inflammation, adaptive immune alterations, pyroptosis, and neuroimmune sensitization that could facilitate CPP chronification. Host-derived damage-associated molecular patterns and metabolic stress appear to be the principal immediate Signal 2 sources, whereas microbiota-derived signals mainly provide priming and indirect contributions. Direct causal evidence in humans remains incomplete, and conflicting microbiome findings may reflect sampling site, disease stage, and methodological differences.

**Discussion:**

The microbiome-inflammasome axis represents one potentially important and testable mechanism in EMs-associated CPP rather than a dominant explanatory framework. Promising therapeutic avenues include probiotics/postbiotics, TLR4 antagonists, NF-κB inhibitors, NLRP3 inhibitors, and IL-1β/IL-18-directed therapies, but most remain exploratory and require validation in prospective, mechanism-based trials. Refining this framework may inform immune stratification and precision immunomodulatory therapy for endometriosis-associated pelvic pain.

## 1 Introduction: the unmet immunological need in endometriosis-associated pain

Endometriosis affects approximately 10% of women of reproductive age globally, and its most prominent clinical manifestation is chronic pelvic pain, which plagues 70%–80% of patients (1, 2). Notably, the severity of CPP often does not correlate with the rASRM stage or lesion size—a clinical paradox suggesting that pain generation is not merely due to mechanical compression or local infiltration but likely involves complex neuroimmune interactions (3, 4). Latent class analysis has reported that different pelvic pain subtypes (e.g., severe cyclic versus severe acyclic pain) are significantly associated with EMs prevalence and multiple chronic overlapping pain conditions, including migraine and low back pain (1, 3, 5), suggesting that central inflammation and immune mechanisms may contribute to pain chronification. Therefore, traditional therapeutic strategies focused solely on lesion excision or hormonal suppression cannot fully alleviate pain, and there is an urgent need to re-evaluate the pathogenesis of EMs-associated CPP from an immunological perspective.

Historically, EMs has been viewed as an estrogen-dependent disease. However, accumulating evidence indicates that immune dysregulation is an important contributor, with widespread disturbances in both innate and adaptive immunity (4, 6). In the peritoneal fluid of EMs patients, multiple immune cell subsets (NK cells, T cells, macrophages) exhibit significant functional and numerical alterations, characterized by impaired immune surveillance and the formation of a locally immunosuppressive microenvironment (7). Myeloid-derived suppressor cells (MDSCs) are markedly elevated in EMs and promote immune evasion and angiogenesis by suppressing the anti-endometriotic activity of T and NK cells (8). Meanwhile, Toll-like receptors, as key pattern recognition receptors of innate immunity, are upregulated on peripheral blood lymphocytes of EMs patients, suggesting that persistent innate immune pathway activation may contribute to local and systemic inflammation (9). Currently, first-line treatments for EMs-associated CPP include hormonal agents and surgical lesion excision (6), but may not adequately correct the immune abnormalities that contribute to pain. Hormonal therapy temporarily suppresses estrogen levels to relieve pain but is accompanied by side effects such as hot flashes and bone loss, with high recurrence rates upon discontinuation (6).Surgical treatment, especially conservative surgery, has a 5-year recurrence rate of 40%–50% (6). Some patients are insensitive to hormonal therapy, and their pain may involve immuno-inflammatory mechanisms beyond estrogen dependence. Hence, there is a pressing need for novel pain management strategies that transcend traditional hormone- and surgery-based approaches and center on immunomodulation.

Microbial imbalance is an important environmental trigger for EMs pathogenesis. Epidemiological evidence indicates that prenatal exposure to endocrine-disrupting chemicals can shorten anogenital distance, thereby increasing the risk of postnatal fecal microbial contamination of the reproductive tract and inducing cervicovaginal dysbiosis (10). MAMPs such as LPS produced by dysbiosis can be recognized by TLR4 on peritoneal macrophages and dendritic cells, activating the MyD88-dependent NF-κB pathway and upregulating NLRP3 inflammasome components (11–13). Notably, a randomized controlled trial found that supplementation with probiotic strains possessing estrogen-metabolizing capacity (Ligilactobacillus salivarius) not only improved pain and quality of life in EMs patients but also reduced serum IL-10 levels, suggesting that microbiota modulation may alleviate pain via effects on the immune–endocrine network (14). IL-1β, released after inflammasome activation, is a key molecule connecting the immune and nervous systems and driving pain chronification. IL-1β can act on IL-1 receptors on nociceptive sensory neurons in the dorsal root ganglia, lowering their activation threshold and triggering peripheral sensitization (15).Simultaneously, IL-1β induces activation of spinal glial cells (microglia and astrocytes), which further release pro-inflammatory cytokines and excitatory neurotransmitters, enhancing synaptic transmission in spinal dorsal horn neurons and leading to central sensitization (15, 16). Therefore, strategies targeting the inflammasome or IL-1β signaling may simultaneously block inflammatory progression and pain sensitization.

Unlike previous reviews that have largely addressed microbiota, inflammasome biology, or neuroimmune pain as separate topics, we propose an integrative “microbiome-inflammasome axis” framework linking dysbiosis to CPP. The core logic is that MAMPs released by dysbiosis of the reproductive or gut tract activate pattern recognition receptors (such as TLRs) on innate immune cells, which in turn trigger NLRP3 inflammasome assembly and activation. In this proposed framework, activated inflammasomes may promote the maturation and release of pro-inflammatory cytokines like IL-1β and IL-18, which not only act directly on pain nerve endings but also induce prostaglandin and nerve growth factor production, leading to peripheral and central sensitization and transforming acute inflammation into chronic, persistent pain. This framework integrates microbiota, immunity, and pain into a coherent conceptual framework, while recognizing that several steps remain experimentally validated only in animal or *in vitro* models and that causal evidence in humans is incomplete. The review is structured as follows: Section 2 summarizes microbial dysbiosis and mucosal immunity; Sections 3 and 4 analyze the LPS-TLR4-NF-κB-NLRP3 cascade; Section 5 examines neuroimmune pain mechanisms; Section 6 discusses therapeutic opportunities and translation; Section 7 presents controversies, alternative mechanisms, and future directions; Section 8 concludes with research priorities.

### 1.1 Methods

This article is a narrative review. Literature was identified by targeted searches of PubMed, Web of Science, and Scopus through (August 2026) using combinations of controlled terms and keywords for endometriosis, chronic pelvic pain, microbiota/dysbiosis, Toll-like receptor 4, NF-kappa B, NLRP3 inflammasome, IL-1beta/IL-18, neuroinflammation, and immunotherapy. Reference lists of relevant reviews and primary studies were screened for additional citations. Studies were included if they addressed mechanisms, clinical associations, or therapeutic interventions relevant to the microbiome-inflammasome axis in endometriosis or related pelvic pain. Because this is a narrative review, no formal risk-of-bias tool or PRISMA flow diagram was used; instead, evidence was appraised by study design, directness, and consistency. Throughout the manuscript, terms such as candidate, potential, may, and associated with are used to distinguish hypotheses or indirect evidence from established causal relationships.

## 2 Microbial dysbiosis: an upstream trigger of immune dysregulation in EMs-associated CPP

### 2.1 Systemic alterations in multi-site microbiota and their impact on mucosal immunity

EMs patients frequently exhibit multi-site dysbiosis that has been associated with altered mucosal immune barrier function. In the gut, dysbiosis is mainly manifested by reduced microbial diversity and a marked decrease in butyrate-producing bacteria (e.g., Lachnospiraceae and Clostridiales) (17), which may impair intestinal barrier integrity and reduce the production of anti-inflammatory short-chain fatty acids (SCFAs) (18). In the reproductive tract, the microbiota from vagina to cervix to uterine cavity and pelvis displays a continuous distribution, and its dysregulation can disrupt local immune homeostasis (10). Critically, the peritoneal fluid microbiome has distinct features; studies have found that peritoneal fluid from patients with deep infiltrating endometriosis is enriched with Flavobacterium, Pseudomonas, and Bacillus (1). Multi-site comparative studies using PERMANOVA analysis reported significant differences in the composition of gut, cervical, and pelvic microbiota (R² = 0.476, P < 0.05), suggesting that dysbiosis at different sites may synergistically promote EMs immunopathology through distinct mechanisms (19–21).

### 2.2 Clinical immunological associations between dysbiosis and CPP phenotypes

Clinical studies have reported associations consistent with a dose-effect relationship between dysbiosis and CPP phenotypes. Reduced gut microbial α-diversity is negatively correlated with higher pain scores (Visual Analogue Scale), and decreased abundance has been associated with more severe pain (17). Specific genera have been identified as potential biomarkers. For example, the abundance of Pseudomonas in peritoneal fluid correlates with pain severity in deep infiltrating endometriosis, and changes in Dorea abundance in the gut are also associated with pain severity (1). As disease stage advances, dysbiosis becomes more pronounced, with enrichment of pro-inflammatory bacteria (e.g., Escherichia coli, Streptococcus) and reduction of anti-inflammatory bacteria (e.g., Bifidobacterium) (17). Regarding causality, current evidence is compatible with a bidirectional relationship: local inflammation and hormonal imbalance caused by EMs lesions can induce dysbiosis; conversely, dysbiosis may exacerbate EMs progression and pain by disrupting mucosal barriers, promoting endotoxin translocation, and activating immuno-inflammatory pathways (21, 22).

### 2.3 Metabolic endotoxemia: a bridge from gut microbiota to pelvic inflammation

A central hypothesis is that dysbiosis contributes to EMs-associated immune activation through the release of pathogen-associated molecular patterns (PAMPs) that initiate innate immune responses. LPS, a major component of Gram-negative bacterial cell walls, is the most representative PAMP. In EMs patients, impaired intestinal barrier function has been associated with translocation of gut-derived LPS into the circulation and peritoneal cavity, with significantly elevated LPS levels in peritoneal fluid that correlate positively with EMs severity (1). This phenomenon, termed “metabolic endotoxemia,” has been proposed as a bridge linking gut dysbiosis to remodeling of the local pelvic immune microenvironment. Elevated LPS is recognized by TLR4 on peritoneal immune cells(especially macrophages), may activate downstream inflammatory pathways (NF-κB and NLRP3 inflammasome) and promote the release of IL-1β and IL-18 (1). Meanwhile, reduced SCFAs resulting from dysbiosis (particularly butyrate deficiency) may diminish their anti-inflammatory and immune-homeostatic effects, leaving LPS-driven pro-inflammatory actions unchecked (18, 20).

### 2.4 Remodeling of the pelvic immune microenvironment: from macrophage polarization to NK cell dysfunction

Dysbiosis may profoundly remodel the pelvic immune microenvironment through persistent PAMP stimulation. At the macrophage level, intestinal barrier disruption and LPS translocation may lead to massive recruitment and aberrant activation of peritoneal macrophages. In EMs, macrophages show enhanced polarization toward the M1 pro-inflammatory phenotype, producing large amounts of reactive oxygen species and pro-inflammatory cytokines (TNF-α, IL-6), while the proportion of M2-type macrophages with anti-inflammatory and tissue-repair functions decreases. This M1/M2 imbalance may contribute to persistent inflammation and lesion progression (23, 24). Macrophage polarization is context- and compartment-dependent: inflamed peritoneal fluid is predominantly M1-skewed, whereas M2-like/tissue-repair macrophages may be relatively enriched in lesions or advanced disease in some studies (24). Similarly, persistent TLR4/NF-κB activation and local antigenic stimulation may promote Th2/Treg-skewed adaptive immunity in some patients, reducing Th1/NK-mediated surveillance and reinforcing lesion immune evasion; direct evidence linking this adaptive shift specifically to the microbiome-inflammasome axis remains limited (24). At the NK cell level, their function is significantly suppressed in EMs patients—animal studies show that although peritoneal NK cell numbers increase in EMs mouse models, their cytotoxic activity declines, impairing the clearance of ectopic endometrial cells and promoting immune evasion and lesion survival (18).

## 3 The LPS–TLR4–NF-κB signaling hub: a candidate regulator of innate immune activation in EMs

### 3.1 Immunological basis of LPS–TLR4 signal initiation in the EMs microenvironment

In the EMs microenvironment, the recognition and binding of LPS to the TLR4/MD-2 complex is considered a potentially important initiating event for innate immune responses. The source of LPS may be related to gut dysbiosis and peritoneal microbiota alterations (25). In addition to exogenous LPS, endogenous danger signals released by cyclic bleeding and necrosis of EMs lesions, such as high-mobility group box 1 (HMGB1) and heat shock protein 70 (HSP70), can also serve as TLR4 ligands to activate innate immunity (11). Notably, estrogen, as a major hormonal driver of EMs, may enhance TLR4 signaling sensitivity, forming a bidirectional “hormone-immune” amplification loop. Estrogen upregulates TLR4 expression or enhances downstream signal transduction, potentially increasing the responsiveness of ectopic lesions to LPS and endogenous ligands, thereby potentially exacerbating local inflammation (25). Conversely, TLR4/NF-κB activation can promote local estrogen synthesis by upregulating aromatase (CYP19A1) and inflammatory mediators such as PGE2, completing the reciprocal arm of this loop; direct evidence in endometriotic tissue remains to be fully established (26, 27). Thus, the initiation of LPS-TLR4 signaling in the EMs microenvironment is not a single event but a complex immunological process co-regulated by microbial products, DAMPs, and sex hormones (**Figure 1**).

**Figure 1:**
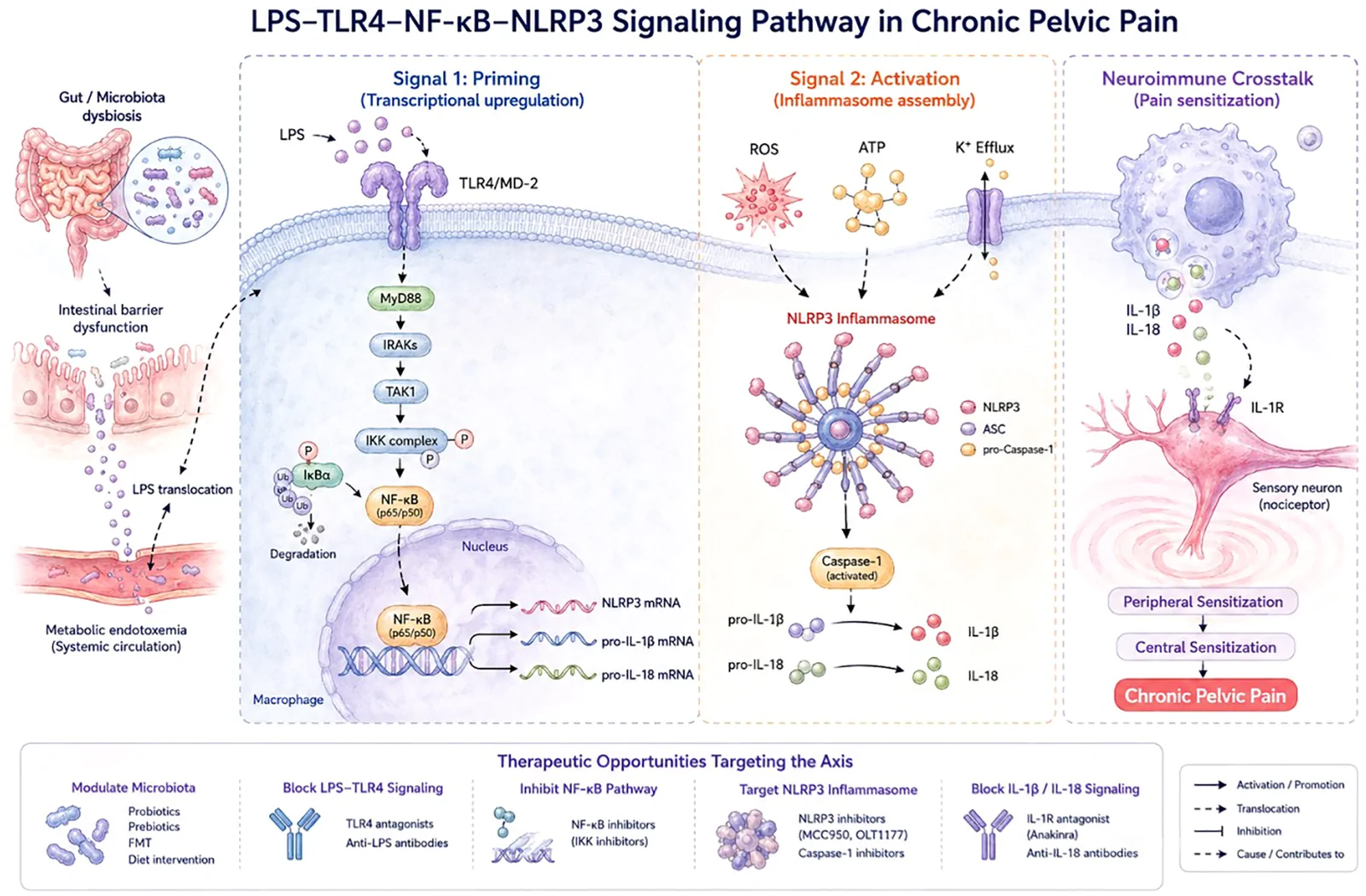
Schematic diagram of the LPS–TLR4–NF-κB–NLRP3 inflammasome signaling cascade. Endometriosis-associated microbiota dysbiosis promotes the release of lipopolysaccharide (LPS) from Gram-negative bacteria, which provides the priming signal (Signal 1). LPS is recognized by the TLR4/MD-2 complex on immune cells (e.g., macrophages), triggering the MyD88-dependent pathway and subsequent NF-κB activation. Upon nuclear translocation, NF-κB upregulates the transcription of NLRP3, pro-IL-1β, and pro-IL-18. Meanwhile, damage-associated molecular patterns (DAMPs) released from ectopic lesions-including ATP (via P2X7 receptor), reactive oxygen species (ROS), and potassium efflux—serve as the activation signal (Signal 2), driving NLRP3 inflammasome assembly with ASC and pro-caspase-1. This leads to caspase-1 activation, cleavage of pro-IL-1β and pro-IL-18 into their mature forms, and subsequent pyroptosis. IL-1β and IL-18 are ultimately released, promoting pelvic inflammation and neuronal sensitization. LPS, lipopolysaccharide; TLR4, Toll-like receptor 4; MD-2, myeloid differentiation factor 2; MyD88, myeloid differentiation primary response 88; NF-κB, nuclear factor kappa-B; NLRP3, NLRP3 inflammasome; ASC, apoptosis-associated speck-like protein containing a CARD; pro-caspase-1, precursor of caspase-1; IL-1β, interleukin-1 beta; IL-18, interleukin-18; DAMP, damage-associated molecular pattern; ROS, reactive oxygen species. Based on current evidence, microbiota-derived LPS mainly provides Signal 1/priming, whereas host-derived DAMPs and metabolic stress are the principal immediate Signal 2 sources; the exact microbiota-versus-host contribution requires further study.

### 3.2 Differential immunological effects of MyD88-dependent and TRIF-dependent pathways

Upon TLR4 activation, signals are transduced mainly through two pathways: the MyD88-dependent pathway and the TRIF-dependent pathway. The MyD88-dependent pathway is the primary route for rapid NF-κB activation, initiating within minutes of ligand binding and driving transcription of large amounts of pro-inflammatory cytokines (IL-1β, TNF-α, IL-6), suggesting an important role in acute inflammation associated with EMs. Studies have shown that in EMs rat models, the expression of TLR4, MyD88, and phosphorylated NF-κB-p65 is significantly upregulated in the spinal cord and dorsal root ganglia, and blockade of MyD88 effectively alleviates mechanical pain (11). In contrast, the TRIF-dependent pathway mediates interferon responses and late-phase inflammation, with later activation kinetics, primarily involved in type I interferon production and dendritic cell maturation.

### 3.3 The NF-κB-driven pro-inflammatory network: from transcriptional regulation to immune effector molecules

NF-κB is a central transcription factor of TLR4 signaling; its nuclear translocation is a pivotal event in the formation of the EMs pro-inflammatory network. Upon activation, NF-κB translocates to the nucleus and binds to κB sites in promoter regions of target genes, initiating transcription of a cascade of pro-inflammatory genes, including IL-1β, IL-6, TNF-α, and cyclooxygenase-2 (28). In the canonical model, NF-κB-mediated transcription serves as the “first signal” for NLRP3 inflammasome activation: it directly upregulates the transcription of NLRP3 protein, pro-IL-1β, and pro-IL-18, providing the essential molecular “raw materials” for inflammasome assembly. Therefore, NF-κB-mediated priming is generally considered a prerequisite for NLRP3 inflammasome activation; even in the presence of second signals (such as ATP or urate crystals), the NLRP3 inflammasome is generally not effectively assembled to produce mature IL-1β in canonical models without NF-κB-mediated transcriptional upregulation. Studies have shown that the TLR4/NF-κB/NLRP3 signaling axis is significantly activated in ectopic lesions and closely correlates with lesion proliferation and angiogenesis (12). Notably, NF-κB can be regarded as the “gatekeeper switch of the NLRP3 inflammasome.” This concept emphasizes the upstream advantage of NF-κB inhibition in blocking the entire inflammatory cascade, while also suggesting potential off-target effects of broad inhibition.

### 3.4 Specific activation of the TLR4/NF-κB pathway in immune cell subsets

The TLR4/NF-κB pathway exhibits distinct activation patterns across different immune cell subsets, collectively shaping the EMs immune microenvironment. In macrophages, TLR4 activation has been implicated in driving polarization toward the M1 pro-inflammatory phenotype (1). In dendritic cells, TLR4 signaling not only promotes their maturation and migration but also upregulates co-stimulatory molecules and MHC molecules via NF-κB, enhancing antigen-presenting capacity. NK cell function is indirectly modulated by the TLR4/NF-κB pathway. This activation pattern is not uniformly unidirectional; downregulation of pattern recognition receptors on macrophages has also been reported in EMs, illustrating stage- and subset-specific complexity (29). Furthermore, stromal cells of ectopic lesions themselves express TLR4 and can respond to LPS or endogenous ligands in an autocrine or paracrine manner; upon NF-κB activation, they may secrete pro-inflammatory cytokines and chemokines, further recruiting and activating immune cells and potentially forming a self-sustaining inflammatory cycle (13, 30).

## 4 The NLRP3 inflammasome: a candidate effector hub for microbiota-driven innate immune activation and pain

### 4.1 The NLRP3 inflammasome as a candidate sensor of innate immunity

The NLRP3 inflammasome is a multi-protein complex composed of NLRP3, ASC, and pro-caspase-1, serving as an important sensor of the innate immune system. NLRP3 expression in EMs lesions is significantly higher than in normal endometrium, indicating aberrant activation (13, 31). Experimental and clinical observations suggest that hyperactivation contributes to EMs pathophysiology by promoting the maturation and release of IL-1β and IL-18, thereby driving inflammatory responses (32). Studies report that NLRP3 inflammasome activation is associated with EMs progression, not only participating in inflammation but also affecting angiogenesis and fibrosis through pyroptosis (33). Thus, the NLRP3 inflammasome is considered a candidate immunological effector hub connecting microbiota-driven signals to CPP, although direct causal evidence in patients remains incomplete.

### 4.2 DAMPs and metabolic stress as NLRP3 second signals

In the canonical model, full NLRP3 inflammasome activation requires two synergistic signals (32, 34): Signal 1 (priming) is mediated by PAMPs (e.g., LPS) via the TLR4/NF-κB pathway, upregulating NLRP3 and pro-IL-1β transcription to provide molecular “raw materials.” Signal 2 (activation) is provided by various DAMPs and metabolic stress signals. In EMs, candidate sources of Signal 2 include ATP released by cyclic bleeding of ectopic lesions (via purinergic receptors such as P2X7 and P2X4), ROS, and potassium efflux (32, 35). Additionally, DAMPs such as urate and cholesterol crystals present in peritoneal fluid, along with local hypoxia and immunometabolic imbalances, may promote NLRP3 inflammasome assembly and activation and sustain chronic inflammation (34). On the basis of current evidence, host-derived DAMPs and metabolic stress are the principal immediate Signal 2 sources, whereas microbiota contribute mainly through LPS-mediated priming, endotoxin translocation, SCFA depletion, and indirect immunometabolic effects. The exact proportion of microbiota- versus host-dependent NLRP3 activation remains unresolved and requires germ-free, antibiotic-treated, or defined-microbiota models.

### 4.3 IL-1β and IL-18: from inflammatory mediators to candidate neuroimmune pain mediators

Upon NLRP3 inflammasome activation, pro-IL-1β and pro-IL-18 are cleaved by caspase-1 into their mature active forms; these cytokines are candidate key molecules linking immune inflammation and pain perception (36). IL-1β has been shown in preclinical models to lower the pain threshold by acting on IL-1 receptors on peripheral nerve endings and may directly sensitize pelvic nociceptors (32, 37), while also inducing prostaglandin and NGF synthesis and potentially stimulating pain signal transmission and maintenance (34). IL-18 may amplify pain signals through effects on neuronal activity in the spinal cord and brain, participating in central pain pathway regulation (38, 39). Therefore, IL-1β and IL-18, as key downstream effectors of the NLRP3 inflammasome, may play direct neuroimmune roles in the development and maintenance of EMs-associated CPP. In addition to their direct neuroimmune actions, IL-1β and IL-18 may shape adaptive immunity by promoting antigen-presenting cell maturation and influencing naïve CD4+ T-cell differentiation. IL-1β can support Th2-polarizing conditions in some experimental systems, whereas IL-18, depending on the local cytokine milieu, may sustain Th1- or Th2/Treg-associated programs. In EMs, persistent NLRP3 activation may therefore contribute to the Th2/Treg-skewed adaptive profiles reported in some patients, although direct human evidence linking inflammasome-derived cytokines to T-cell subset skewing remains limited; this mechanism should be regarded as a candidate adaptive immune consequence of the microbiome-inflammasome axis.

### 4.4 Immunological clinical evidence for NLRP3 inflammasome activation and CPP severity

Clinical and observational studies provide supportive evidence linking NLRP3 inflammasome activation to EMs-associated inflammation. NLRP3 activation-related markers are associated with EMs diagnosis and immune characteristics (40). Elevated NLRP3 pathway components and IL-1β/IL-18 have also been reported in reproductive-tract and follicular samples from women with EMs (41). These findings support, but do not yet establish, a central role of NLRP3 inflammasome activation and its downstream products in EMs-associated CPP pathophysiology, and they provide a rationale for further mechanistic and therapeutic studies targeting the NLRP3 inflammasome.

## 5 Neuroimmune interactions: from peritoneal inflammation to central pain sensitization

A mechanistic bridge between peritoneal TLR4/NLRP3 activation and central sensitization involves circulating pro-inflammatory cytokines (IL-1β, TNF-α) and LPS, retrograde afferent signaling from pelvic organs to the dorsal root ganglia (DRG), DRG satellite glial activation, and spinal microglial/astrocytic responses. These pathways can transfer peripheral innate immune activation to the spinal cord and DRG, where TLR4 is upregulated and contributes to central sensitization (37, 39, 42).

### 5.1 Peripheral sensitization: direct effects of inflammatory mediators on pelvic nociceptors

In EMs, inflammatory mediators in the pelvic microenvironment can act directly on sensory neurons and may contribute to peripheral sensitization, a proposed initial step in CPP development. Pro-inflammatory cytokines such as IL-1β and TNF-α bind to their receptors on sensory neurons, lowering activation thresholds and enhancing responsiveness to noxious stimuli (15, 37, 42). Meanwhile, PGE2 produced by upregulated COX-2 is a recognized mediator of hyperalgesia and can sensitize primary afferent nerve endings through EP receptors (3). Elevated NGF in the inflammatory environment may not only directly enhance nociceptor excitability but also induce new sensory nerve innervation around ectopic lesions (2).

### 5.2 Central sensitization: neuroinflammation and central plasticity changes

When peripheral nociceptive input from the pelvis persists, plastic changes occur in the central nervous system—central sensitization—which is widely considered a core mechanism underlying CPP chronification and generalization. At the spinal cord level, repetitive noxious stimulation can lead to hyperexcitability of dorsal horn neurons, accompanied by activation of central immune cells (particularly microglia and astrocytes), which may release large amounts of pro-inflammatory cytokines, BDNF, and glutamate, further increasing neuronal excitability and potentially forming a neuron–glia positive feedback loop (15, 16, 39). Persistent neuroinflammation may lead to altered electrophysiological activity in pain-related brain regions, marking global functional remodeling of the central pain processing network (16, 43).

### 5.3 Immunoregulatory role of the gut–brain axis in EMs-associated pain

The gut-brain axis may play an important immunoregulatory role in EMs-associated pain. EMs patients frequently exhibit gut dysbiosis, which may disrupt the intestinal barrier, allowing bacterial products (e.g., LPS) to enter the circulation, potentially triggering systemic inflammation and activating immune cells. Simultaneously, chronic pain, as a stressor, activates the HPA axis, leading to altered cortisol secretion patterns (**Figure 2**). Studies (44)have shown that in EMs patients with CPP, pain severity correlates with blunted HPA axis responsiveness to CRH, manifested by blunted cortisol and ACTH release responses, reflecting long-term HPA axis exhaustion or maladaptation. Pain-related psychological distress is also common in this population (45). HPA axis dysfunction may interact with gut dysbiosis, further exacerbating neuroinflammation and central sensitization and potentially forming a vicious cycle. Mechanistically, altered cortisol/CRH signaling can reduce intestinal motility, alter mucus and IgA secretion, increase mucosal permeability, and shift microbial composition, thereby completing the pain-to-dysbiosis feedback arm of this cycle.

**Figure 2:**
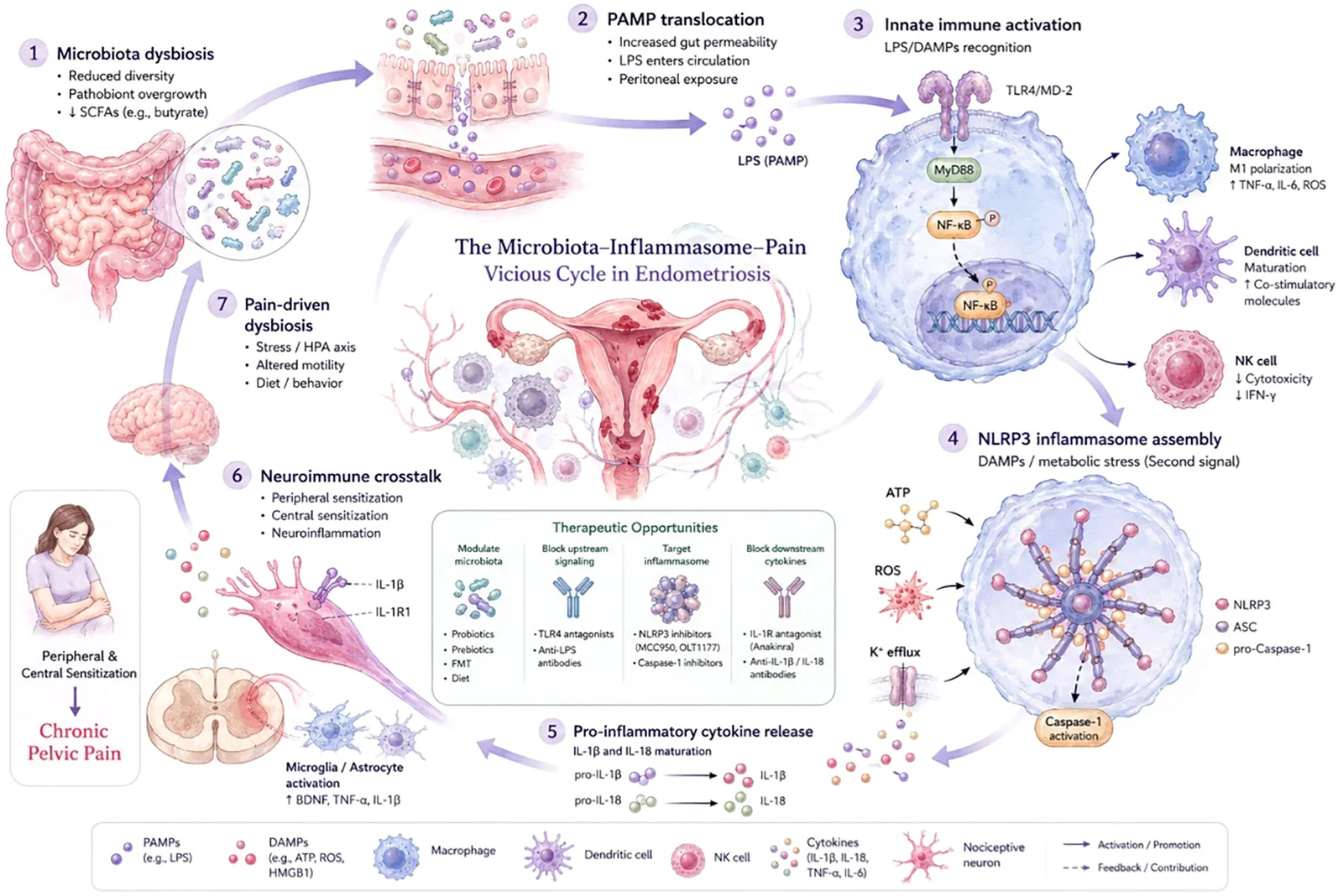
Conceptual model of the microbiota–inflammasome–pain positive feedback loop in endometriosis-associated chronic pelvic pain. This schematic illustrates the self-perpetuating circuit linking gut/reproductive tract dysbiosis, innate immune activation, and chronic pelvic pain. Step 1: Microbiota dysbiosis may increase lipopolysaccharide (LPS) levels, which may activate the TLR4/NF-κB pathway and promote NLRP3 inflammasome assembly via the dual-signal mechanism (Signal 1: LPS/TLR4/NF-κB-mediated transcriptional priming; Signal 2: DAMPs-induced inflammasome activation). Step 2: NLRP3 inflammasome activation leads to the maturation and release of IL-1β and IL-18. Step 3: These pro-inflammatory cytokines directly sensitize pelvic nociceptors and induce prostaglandin and nerve growth factor synthesis, and may contribute to peripheral and central sensitization that culminates in chronic pelvic pain. Step 4: Pain, as a chronic stressor, further disrupts microbiota homeostasis via the hypothalamic–pituitary–adrenal (HPA) axis, potentially creating a positive feedback loop that reinforces dysbiosis and sustains inflammation (dysbiosis → inflammation → pain → dysbiosis). Key immune cells (macrophages and NK cells) and molecular components (PAMPs, DAMPs, inflammasome assembly, and neuroimmune interactions) are indicated. LPS, lipopolysaccharide; TLR4, Toll-like receptor 4; NF-κB, nuclear factor kappa-B; NLRP3, NLRP3 inflammasome; IL-1β, interleukin-1 beta; IL-18, interleukin-18; DAMP, damage-associated molecular pattern; PAMP, pathogen-associated molecular pattern; NK, natural killer; HPA, hypothalamic–pituitary–adrenal. Step 7 is supported by neuroendocrine mechanisms: chronic pain activates the HPA axis, and altered cortisol/CRH responses can affect intestinal permeability, motility, mucosal immunity, and microbiota composition.

## 6 Therapeutic opportunities: immunotherapeutic strategies targeting the microbiome–inflammasome axis

### 6.1 Targeting the microbiome: restoring mucosal immune homeostasis

Microbiome dysregulation is considered a plausible upstream trigger of EMs immunopathology, so restoring mucosal immune homeostasis is a promising but still exploratory strategy. Probiotic supplements, especially Lactobacillus strains capable of metabolizing estrogen, show potential for modulating immune and hormonal microenvironments. A randomized double-blind trial (14)showed that oral Ligilactobacillus salivarius CECT 30632 significantly reduced serum estradiol (≈50%) and improved pain, emotional well-being, and quality of life, with concomitant decreases in IL-10. In an assisted-reproduction setting, probiotic use has also been explored in women with EMs-associated infertility (46). Mechanistically, gut microbial β-glucuronidase activity and estrobolome dysregulation can increase estrogen deconjugation and enterohepatic recycling, thereby raising local estrogen bioavailability; probiotics capable of metabolizing estrogens may reduce this microbial contribution (47, 48). Broad-spectrum antibiotics remodeling the vaginal microbiota may alleviate local inflammation and immune activation by eliminating pathogens and restoring a Lactobacillus-dominant structure, although their long-term effects remain unclear. Fecal microbiota transplantation (FMT) in EMs remains in early exploration; experimental studies suggest that gut microbiota-derived signals can promote IL-17A-mediated lesion chemotaxis, although human data remain limited (49). Dietary interventions (e.g., low-FODMAP or anti-inflammatory diets) can modulate gut microbiota composition and metabolites (SCFAs), indirectly affecting mucosal immune function (20). Because the causal direction of dysbiosis remains uncertain, microbiome-targeted approaches should currently be regarded as exploratory adjuncts requiring randomized controlled trials with mechanistic endpoints.

### 6.2 Targeting upstream and downstream factors of innate immune signaling: precision immune intervention

**Table 1** summarizes representative preclinical immunotherapeutic interventions targeting the microbiome-inflammasome axis. The inflammasome may serve as a central platform connecting upstream dysbiosis to downstream inflammatory effectors, making it a target for precision intervention. In preclinical models, TLR4 antagonists have been shown to block upstream signals, suppress downstream pro-inflammatory cytokine production, and exert anti-inflammatory effects. NF-κB inhibitors (e.g., curcumin) have demonstrated anti-inflammatory and analgesic potential in EMs studies, effectively reducing TNF-α, IL-6, and IL-1β expression in ectopic lesions (50). Parthenolide has been shown to inhibit NLRP3 activation and reduce IL-1β and IL-18 maturation and release by activating the AMPK/ULK1 autophagy pathway, promoting autophagy of damaged mitochondria and NLRP3 components, thereby achieving anti-inflammatory and anti-proliferative activity (51). Given that IL-1β is a key downstream effector of NLRP3, the IL-1 receptor antagonist (anakinra) has been successfully used in autoimmune inflammatory diseases such as rheumatoid arthritis, and its application in EMs may hold promise by blocking IL-1 signaling to suppress local and systemic inflammatory cascades and alleviate CPP.

**Table 1:** Summary of preclinical immunotherapeutic interventions targeting the microbiome–inflammasome axis.

Drug/Intervention	Target	Animal model	Major immunological effects	Limitations
Parthenolide (PTL)	NLRP3 inflammasome + AMPK/ULK1 autophagy	C57BL/6 mice, intraperitoneal implantation EMs model (n=30)	① 10 mg/ml PTL for 1 h *in vitro* significantly inhibited NLRP3, caspase-1-p20, IL-1β, and GSDMD activation; ② increased Beclin1 and AMPK/ULK1 phosphorylation, decreased p62 and LC3, enhanced autophagic flux; ③ intraperitoneal PTL significantly reduced lesion number, surface area, and weight, and inhibited inflammatory protein expression in lesions (51)	① High concentration (10 mg/ml) used *in vitro*; optimal *in vivo* dosing not systematically optimized; ② intraperitoneal administration limits clinical translation
MCC950	NLRP3 inflammasome (specific inhibitor)	Mouse ovarian endometriosis (OE) model	① Significantly reduced ovarian ectopic lesion volume (89 ± 15 vs. 49 ± 9.3 mm³/ovary; P < 0.05); ② inhibited cyst-derived stromal cell (CSC) survival and IL-1β secretion; ③ reduced IL-1β and Ki67 in lesions, improved granulosa cell oxidative stress markers (31)	① Low oral bioavailability; ② long-term systemic NLRP3 inhibition may increase infection risk
TAK-242 (Resatorvid)	TLR4 (specific inhibitor)	C57BL/6 mice (wild-type + TLR4^−^/^−^), EMs model	① Inhibited LPS/ATP-induced NF-κB/NLRP3 activation, pyroptosis, and IL-1β secretion (P < 0.05); ② TLR4 knockout or TAK-242 treatment significantly reduced lesion weight, PCNA proliferation marker, and CD31 microvessel density (all P < 0.05); ③ blocked NF-κB nuclear translocation and downstream inflammasome activation (12)	① Systemic TLR4 inhibition may affect normal immune surveillance; ② long-term safety needs evaluation
1,25-Dihydroxyvitamin D_3_	NF-κB pathway (via VDR activation and IκBα stabilization)	Rat EMs model	① Significantly reversed LPS-induced proliferation, migration, and inflammatory cytokine production in ectopic endometrial stromal cells (EESCs) and Ishikawa cells, induced apoptosis; ② inhibited phosphorylated p65 expression and nuclear translocation in LPS-activated cells; ③ significantly inhibited lesion growth *in vivo*, suppressed NF-κB pathway activation and associated inflammatory phenotype (30)	① Immunomodulatory effects are dose-dependent; optimal therapeutic window unclear; ② long-term high-dose vitamin D may cause hypercalcemia
Curcumin	NLRP3 inflammasome + oxidative stress	Mouse EMs model	① Significantly reduced adhesion scores, lesion number and volume; ② reduced oxidative stress; ③ inhibited pyroptosis-related proteins including NLRP3, caspase-1, GSDMD, IL-1β, and IL-18 (50)	① Extremely low oral bioavailability; ② needs nanoformulations to improve pharmacokinetics
Probiotics (*Lactobacillus gasseri* OLL2809)	NK cell activation + microbiome modulation	Mouse EMs model	① Inhibited intraperitoneal ectopic endometrial cell development by activating NK cells; ② stimulated IL-12 production in mouse splenocytes (56)	① Strain-specific; efficacy varies greatly; ② multi-target, causal validation difficult
Resveratrol	Immune dysregulation + oxidative stress	Endometriotic cell–macrophage co-culture	① Targeted immune dysregulation and oxidative imbalance in co-culture; ② modulated inflammatory microenvironment (57)	① Low oral bioavailability; ② multi-target, insufficient mechanistic specificity

Evidence level: all interventions summarized in **Table 1** are preclinical (*in vitro*, animal, or ex vivo); no clinical trial evidence is available for these specific interventions.

### 6.3 Translational barriers and solutions for immunotherapy

Although EMs immunotherapy shows great potential in animal models, multiple barriers remain for clinical translation (52). The primary challenge is species differences between mouse and human immune systems (6). Second, route of administration and bioavailability are critical; many immunomodulators have low oral bioavailability. Developing novel drug delivery systems such as nanoparticles or sustained-release implants is a potential direction. Finally, long-term safety of immunosuppression is crucial; systemic NLRP3 or IL-1β inhibition may increase infection risk, necessitating more precise local action strategies to balance efficacy and safety. For clinical positioning, recent systematic evidence supports GnRH antagonist regimens with add-back therapy and post-operative dienogest for pain and recurrence, underscoring that even established hormonal options need individualized sequencing; microbiome-inflammasome-targeted strategies should therefore be evaluated against these current standards (53, 54).

### 6.4 Combination therapeutic strategies based on immune stratification

Given the complexity of EMs immunopathology, single-target strategies may have limited efficacy, and combination strategies based on immune stratification are promising but still exploratory. (i) Select targets according to immune activation stage: patients in early stages with predominant upstream dysbiosis and TLR4 hyperactivation may preferentially receive probiotics or TLR4 antagonists; patients in middle-to-late stages with NLRP3 inflammasome activation and IL-1β release as prominent features may preferentially receive downstream inhibitors like MCC950 (55). (ii) Individualize treatment according to pain phenotype: anti-inflammatory immunotherapy may be more effective for predominantly inflammatory pain; patients with neuropathic pain may need combination with neuromodulators. The immunological rationale for combination therapy (11, 31, 51) is that antibiotics or probiotics may clear upstream triggers (e.g., pathogenic bacteria), thereby reducing persistent NLRP3 activation signals, while inflammasome inhibitors may block downstream effects (e.g., IL-1β release), potentially forming a synergistic “upstream clearance + downstream blockade” that may break the inflammatory vicious cycle while reducing the dosage and side effects of single drugs used long-term. However, given the unresolved causal relationship between dysbiosis and EMs (21), these proposals should be tested in prospective trials with microbiome, immune, and pain endpoints.

## 7 Controversies, open questions, and future directions

### 7.1 Controversy 1: is dysbiosis a “cause” or “consequence” of EMs?

The causal relationship between dysbiosis and EMs remains the most controversial core question. Evidence supporting “cause” comes mainly from animal studies, where antibiotic intervention significantly reduces EMs lesion establishment and growth; evidence supporting “consequence” also comes from observational and experimental studies, as local chronic inflammation from EMs lesions can reciprocally shape peritoneal fluid and gut microbiota composition (1). Overall, dysbiosis and EMs may form a positive feedback loop: initial immune dysregulation or genetic susceptibility may lead to microbial imbalance, which may exacerbate inflammation via the TLR4/NLRP3 pathway and the inflammation further disrupts microbial homeostasis; recent integrative reviews emphasize this bidirectional framework (21).

In addition, reports differ on which genera are pathogenic or protective. Some studies emphasize reduced Lactobacillus and increased Gardnerella in the reproductive tract, whereas others report enrichment of Pseudomonas, Flavobacterium, and Bacillus in peritoneal fluid; still others emphasize reduced gut diversity, loss of SCFA-producing taxa, or functional changes such as metabolic endotoxemia. These discrepancies may reflect sampling site, disease stage, sequencing methods, and covariates including hormonal status, diet, and prior antibiotic use. Community-level function and site-specific microbial niches may therefore be more robust than any single genus as a biomarker (17, 19–22).

### 7.2 Controversy 2: is the NLRP3 inflammasome a “driver” or “bystander” of pain?

The role of the NLRP3 inflammasome in EMs-associated CPP is also under debate. Evidence supporting the “driver” role comes from studies of NLRP3 activation-related markers (IL-1β, LY96, PDIA3) in EMs endometrium (40). However, much of the evidence remains associative or indirect, and pain can also involve stress-related glial activation and neuroimmune mechanisms independent of NLRP3 (58). Longitudinal validation using TLR4 or NLRP3 gene knockout mouse models is urgently needed. Until such evidence is available, the NLRP3 inflammasome should be viewed as one potentially important mechanism rather than the sole driver of CPP.

### 7.3 Alternative and parallel pathogenic mechanisms

Endometriosis is a highly heterogeneous disease involving endocrine, neurogenic, genetic, epigenetic, vascular, and additional immune mechanisms (4, 6, 25, 26, 59). The TLR4/NF-κB/NLRP3 axis should therefore be regarded as one potentially important and testable mechanism rather than the dominant explanatory framework. Notably, TLR4/NF-κB/NLRP3 signaling can be protective during acute infection and tissue repair, whereas persistent low-grade LPS exposure, SCFA deficiency, estradiol-mediated sensitization, and maladaptive neuroimmune amplification can shift the same pathway toward chronic pelvic inflammation and pain. Future studies should define the timing, compartment, ligand repertoire, and counter-regulatory mechanisms that determine whether activation of this axis is protective or pathological (15, 32).

### 7.4 Protective versus pathological TLR4/NF-κB/NLRP3 signaling

The same signaling cascade can be protective or pathological depending on timing, dose, ligand repertoire, and tissue context. In acute infection, TLR4/NF-κB activation and NLRP3-mediated IL-1β/IL-18 production are essential for host defense and tissue repair; NLRP3-dependent responses are critical for controlling pathogens, and transient NF-κB activation coordinates antimicrobial peptides, neutrophil recruitment, and resolution of inflammation (34). In contrast, in EMs, persistent low-grade LPS exposure, DAMPs released by cyclic bleeding, SCFA depletion, estrogen sensitization, and maladaptive neuroimmune amplification may convert this pathway into a chronic inflammatory amplifier. Preclinical examples include TLR4 knockout or TAK-242-mediated TLR4 inhibition reducing lesion growth, proliferation, and angiogenesis (12); MCC950 reducing ovarian endometriotic lesion volume and IL-1β secretion (31); and 1,25-dihydroxyvitamin D3, curcumin, or parthenolide suppressing NF-κB/NLRP3 activation and pyroptosis in EMs models (30, 50, 51). Clinical evidence remains largely observational, such as increased NLRP3-associated markers and IL-1β/IL-18 in reproductive-tract or follicular samples (40, 41). Future work should therefore define the timing, compartment, ligand repertoire, and counter-regulatory mechanisms that determine whether this axis is protective or pathological.

### 7.5 Safety and ethical considerations of immunotherapy

Immunotherapy targeting the microbiota-inflammasome axis, while promising in preclinical models, is accompanied by non-negligible safety and ethical challenges. Long-term NLRP3 inhibitors may increase infection risk because NLRP3 is critical for host defense against many pathogens; systemic inhibition may impair innate immunity. Microbiome-remodeling interventions such as antibiotics or FMT may have profound effects on the gut ecosystem (17, 22). The ideal strategy should focus on local or targeted interventions, developing NLRP3 inhibitors that target the peritoneal microenvironment or using probiotics to deliver anti-inflammatory factors, to minimize systemic side effects.

### 7.6 Future directions

To break current research bottlenecks, future studies should focus on:

First, single-cell sequencing combined with spatial transcriptomics to resolve the specific activation patterns of the TLR4/NF-κB/NLRP3 pathway in different immune cell subsets and clarify their spatial distribution in EMs lesions.

Second, large-scale prospective cohort studies collecting microbiota composition, immune markers, and pain phenotype data to validate causal associations between microbial/immune markers and pain progression.

Third, integration of organoids and artificial intelligence to construct humanized immune microenvironment models using patient-derived organoids co-cultured with immune cells, combined with AI for high-throughput drug screening, accelerating mechanistic validation and new target discovery.

Fourth, combination intervention clinical trials evaluating the efficacy and safety of antibiotics, probiotics, NLRP3 inhibitors, or botanical/traditional Chinese medicine interventions in combination (60, 61).

Fifth, multi-omics integration through metagenomics, metabolomics, and immune repertoire sequencing to systematically reveal the panoramic network of microbiota–host immune interactions, providing a basis for precise subtyping and individualized treatment of endometriosis.

## 8 Conclusion

The microbiome-inflammasome axis is supported by convergent preclinical and observational evidence as a promising, but not yet causally established, framework connecting dysbiosis and CPP. The TLR4/NF-κB/NLRP3 axis, by linking innate immune recognition, inflammasome activation, and neuroimmune sensitization, may contribute not only to EMs lesion progression but also to pain chronification. This framework expands the pathological understanding of EMs pain from purely hormone-dependent inflammation to persistent immune responses to microbial signals, while acknowledging that direct causal proof in humans remains incomplete.

Given the heterogeneity of EMs, this axis should be interpreted as one potentially important mechanism rather than the dominant explanatory framework. Genus-level differences and conflicting findings should be interpreted in light of sampling site, disease stage, and methodology. TLR4/NF-κB/NLRP3 activation can be protective in acute inflammation but may become maladaptive in chronic EMs; future interventions should therefore aim to restore immune homeostasis rather than simply block the pathway.

Future research should validate the spatiotemporal activation of this axis in human samples, develop non-invasive biomarkers based on serum or peritoneal fluid NLRP3-related products, and test individualized immunotherapy in prospective trials that integrate microbiota, immune, and pain outcomes.
